# A Novel Neuropsychological Tool for Immersive Assessment of Concussion and Correlation with Subclinical Head Impacts

**DOI:** 10.1089/neur.2020.0022

**Published:** 2021-05-26

**Authors:** Tamara R. Espinoza, Kristopher A Hendershot, Brian Liu, Andrea Knezevic, Breanne B. Jacobs, Russell K. Gore, Kevin M. Guskiewicz, Jeffery J. Bazarian, Shean E. Phelps, David W. Wright, Michelle C. LaPlaca

**Affiliations:** ^1^ Department of Emergency Medicine, Division of Emergency Neurosciences, Emory University School of Medicine, Atlanta, Georgia, USA.; ^2^ Georgia Tech Research Institute (GTRI), Advanced Human Integration Branch, Atlanta, Georgia, USA.; ^3^ Department of Biostatistics and Bioinformatics, Rollins School of Public Health, Emory University, Atlanta, Georgia, USA.; ^4^ Complex Concussion Clinic, Shepherd Center, Atlanta, Georgia, USA.; ^5^ Department of Exercise and Sport Science, University of North Carolina, North Carolina, USA.; ^6^ Department of Emergency Medicine, University of Rochester, Rochester, New York, USA.; ^7^ Department of Biomedical Engineering, Georgia Institute of Technology/Emory University, Atlanta, Georgia, USA.

**Keywords:** concussion, helmet impact sensor, mild traumatic brain injury, neuropsychological test, subconcussive impact

## Abstract

Mild traumatic brain injury (mTBI) remains a diagnostic challenge and therefore strategies for objective assessment of neurological function are key to limiting long-term sequelae. Current assessment methods are not optimal in austere environments such as athletic fields; therefore, we developed an immersive tool, the Display Enhanced Testing for Cognitive Impairment and mTBI (DETECT) platform, for rapid objective neuropsychological (NP) testing. The objectives of this study were to assess the ability of DETECT to accurately identify neurocognitive deficits associated with concussion and evaluate the relationship between neurocognitive measures and subconcussive head impacts. DETECT was used over a single season of two high school and two college football teams. Study participants were instrumented with Riddell Head Impact Telemetry (HIT) sensors and a subset tested with DETECT immediately after confirmed impacts for different combinations of linear and rotational acceleration. A total of 123 athletes were enrolled and completed baseline testing. Twenty-one players were pulled from play for suspected concussion and tested with DETECT. DETECT was 86.7% sensitive (95% confidence interval [CI]: 59.5%, 98.3%) and 66.7% specific (95% CI: 22.3%, 95.7%) in correctly identifying athletes with concussions (15 of 21). Weak but significant correlations were found between complex choice response time (processing speed and divided attention) and both linear (Spearman rank correlation coefficient 0.262, *p* = 0.02) and rotational (Spearman coefficient 0.254, *p* = 0.03) acceleration on a subset of 76 players (113 DETECT tests) with no concussion symptoms. This study demonstrates that DETECT confers moderate to high sensitivity in identifying acute cognitive impairment and suggests that football impacts that do not result in concussion may negatively affect cognitive performance immediately following an impact. Specificity, however, was not optimal and points to the need for additional studies across multiple neurological domains. Given the need for more objective concussion screening in triage situations, DETECT may provide a solution for mTBI assessment.

## Introduction

Mild traumatic brain injury (mTBI) and concussion have gained increasing attention and notoriety within the medical and lay communities over the last decade. Despite state-initiated legislation for youth sports injury mitigation, and widespread rule changes aimed at reducing the number and magnitude of head impacts, sports related concussion (SRC) comprises a significant number of sports injuries overall.^1^ Between 2010 and 2016, there were an average of 283,000 emergency department (ED) visits per year among children for SRC.^2^ Considering over 8 million high school students participate in sports, and nearly 500,000 students participate in the National Collegiate Athletics Association (NCAA) annually,^3^ student athletes are notably at risk for concussion.

The effects of repeated concussions are recognized as a potential major contributor to long-term disability,^4–9^ including growing evidence of persistent cognitive, functional, and psychological effects,^10–12^ prompting efforts to more accurately identify concussions and prevent or limit future concussions. However, the initial identification of individuals with potential concussive brain injury remains a diagnostic challenge, especially in real or near real-time. In an effort to better identify potentially concussed individuals immediately after head injury, standard symptom checklists and dynamic physical assessments have been utilized, with varying degrees of success.^13,14^

Currently, the Sport Concussion Assessment Tool (SCAT), 5th edition, is recommended for assessment in the first 24 h following concussion.^15^ The SCAT has excellent sensitivity and specificity, yet requires a trained healthcare professional for administration and interpretation.^16^ Although the role of standardized neuropsychological (NP) testing as a part of a comprehensive approach to concussion management and return-to-play decisions is recommended,^15^ current NP assessment tools typically require test administration in quiet or calm environments to improve the validity and sensitivity of test results.^17^ These restrictions limit the utility and feasibility of NP testing in field or near point of injury environments, and hinder the capability for decision support to clinical staff during a game or event, although consideration of NP testing in EDs has been considered,^18^ lending support for on-field shortened test formats. Other computerized testing platforms such as Immediate Post-Concussion Assessment and Cognitive Testing (ImPACT) are not designed for sideline assessing and the ImPACT Quick-Test (a 5–7 min mobile test) is not yet validated.

Although the links between concussion history and chronic effects are still an active area of research, perhaps the risk of neurological impairment as a result of cumulative head impact exposures without a clinical diagnosis of concussion is even less understood.^19,20^ The use of helmet impact sensors enables investigation of the relationship between head impact parameters and neurocognitive function. Some studies report no significant associations^21,22^ and others find significant correlations among impact exposure and cognitive deficits.^23,24^ Given the differences in methodology and analyses among concussions studies, relationships between impact factors and cognitive function deserve more attention.

To address the need for tools for near real-time decision support and sideline assessment of athletes with suspected concussion, we developed Display Enhanced Testing for Cognitive Impairment and mTBI (DETECT), which leverages a heads-up display and noise reduction headphones to create an immersive platform for shortened NP testing. The aims of this study were: 1) to test the feasibility and the sensitivity of DETECT as an acute sideline NP assessment tool for concussion (mTBI) injuries in a cohort of competitive football athletes, and 2) to examine the relationship between helmet impact acceleration and immediate NP performance in football athletes with no signs of concussion.

## Methods

### Participant selection

Football players from the University of North Carolina (Chapel Hill, NC; NCAA Division 1), the University of Rochester (Rochester, NY; NCAA Division 3) and two local Division AAA high schools in metro Atlanta, GA, were recruited for participation. Athletes were eligible for participation if they were an active member of the football team during the testing season, and were 16 years of age or older. Students were excluded if they were not fluent in English, reported a baseline neurological disability (seizures, modified Rankin score >1), had a concussion within the previous 6 months, or demonstrated poor effort on baseline DETECT testing. Athletes younger than 18 years of age required parental consent (and athlete assent) prior to study enrollment. This study was approved by the funding sponsor's Human Research Protections Office, the Emory University and Georgia Tech Institutional Review Boards (IRBs), as well as the IRB at each collegiate study site.

### DETECT platform

**FIG. 1.**
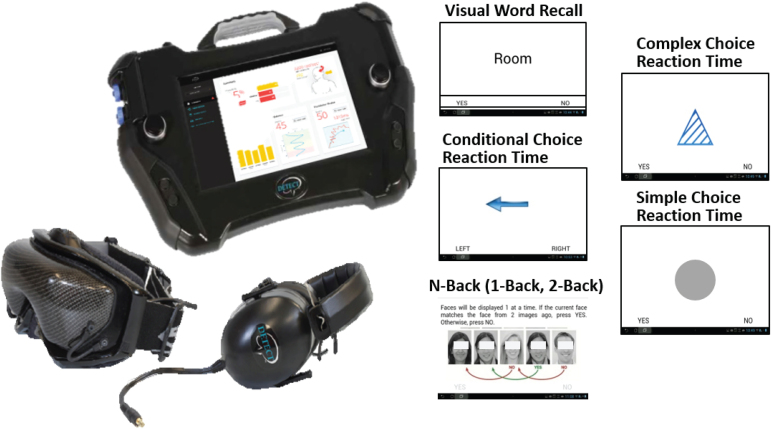
The DETECT platform and user interface. The main unit is an Android tablet housed in a custom, ruggedized case with two input buttons for binary responses. A custom, heads-up display has interpupillary and focus adjustment. Noise attenuation headphones have pink noise and allow for instruction delivery. Subtest sample prompts shown. Eyes obscured in figure only. DETECT, Display Enhanced Testing for Cognitive Impairment and mTBI; mTBI, mild traumatic brain injury.

The DETECT system comprises a heads-up display visor and noise-reducing headphones, along with a handheld platform for test battery administration, subject response inputs, and data output (Fig. 1). These features effectively provide an immersive environment for NP assessment in remote, noisy, or distracting environments.^25^ The DETECT NP assessment was developed by modifying elements of a battery of standard pencil-and-paper NP tests that, in their full form, have been previously validated for mTBI.^26^

The NP battery consists of a series of five, short NP subtests with multiple trials each that evaluate information processing speed, working memory, and executive function. Specifically, the battery includes: 1) visual word recall (working memory, learning, and recall) with three trials following presentation of 12 words to remember; each trial consists of 24 words (12 original and 12 matched distractors) individually presented; two consecutive rounds with selective reminding in the second, and a trial at the end of the battery for delayed reminding; subject selects “yes” or “no” if word is in original list of 12; 2) conditional choice reaction time (processing speed and divided attention) with arrows in which subject selects “right” or “left” where blue points to correct side and red points to opposite side; 3) N-back (1-back, working memory, processing speed; 2-back, working memory with increased load) in which subject selects “yes” or “no” to indicate if they saw the face in the previous prompt (1-back) or two previous (2-back); 4) complex choice reaction time (processing speed and divided attention) with colored shapes for which subject selects “yes” or “no” if shape, color, and pattern match reference shape; and 5) simple choice reaction time (processing speed), which is similar to the complex choice test but with a simple shape only, serving as an effort test to gauge poor performance due to inattention or poor effort.

DETECT takes approximately 10 min to complete. A final score as well as individual subtest performance (with response time, accuracy, time to completion, and non-entry) are generated. The final score is within a proprietary scale ranging from 1 to 10 (with higher scores indicative of worsening cognitive function) that incorporates the individual subtests and reflects the probability of impairment. The algorithm for the score was derived from a multi-variable predictive ordinal regression model and validated using a 10-fold cross-validation approach in a cohort of 405 subjects with mild cognitive impairment (MCI) and aged-matched controls (median age 77.9 years), against a 90-min formal NP test battery, 10-item Functional Assessment Questionnaire,^27^ and professional neuropsychologist judgement.^28^ DETECT also performed superiorly to the Mini Mental State Examination (MMSE; c-index 0.995 vs. 0.901) in identifying MCI.^28^ In a separate validation study, DETECT was tested in 39 subject who were HIV-positive (median age 48 years) and compared with an eight-part NP battery with moderate to high predictive ability (Spearman's coefficient 0.59, *p* < 0.0001).^29^ Neither DETECT normative scores nor validation in the adolescent and young adult population is available at this time.

### Helmet impact sensors

All players participating in the study were instrumented with Riddell HIT (Head Impact Telemetry; Simbex, Lebanon, NH, USA) helmet accelerometers for detecting linear and rotational acceleration at impact and location of impact (version 1) similar to previous reports.^30^ The system default impact registration cutoff was 14.4*g*. HIT has a built-in algorithm to minimize false-positive impacts.^31^ We randomly selected four games from accessible, complete video footage and manually compared impacts that registered on the HIT system with actual impacts.

During impact practices throughout the season we monitored in real time and flagged players who registered an impact with combinations of translational and rotational accelerations impact criteria (30–60*g* and <3000 rad/sec^2^, 61–90*g* and <3000 rad/sec^2^, >91*g* and <3000 rad/sec^2^, 30–60*g* and >3000 rad/sec^2^, 61–90*g* and >3000 rad/sec^2^, >91*g* and >3000 rad/sec^2^), such that each of the six acceleration combinations, or bins, had at least 10 players. Flagged players were tested on the sidelines with the complete DETECT battery within 15 min of the qualifying impact and could not be retested within 2 weeks of qualifying. To qualify, players also could not have had sustained a linear or rotational acceleration (archived by the HIT system) in the previous 2 weeks that was greater than the current acceleration combination for which they were being tested. In addition, subjects did not qualify for testing if they had taken a DETECT test (for any reason) within the previous 7 days.

### Assessment parameters

Enrolled athletes completed in the training room a screening questionnaire followed by the DETECT test assessment prior to the beginning of the football season. This evaluation served as a baseline test for comparison with future DETECT assessments. To reduce the learning effect of repeated assessments, the DETECT battery is designed to pull from a random selection of words, shapes, and stimuli during each test administration.

Throughout the season, any athlete suspected of sustaining a concussion was removed from play and completed sidelines clinical concussion screening by the on-site athletic trainer (AT) or team physician, who were not involved in the study and did not know the results of the DETECT tests. Following their school assessment, athletes were administered DETECT. When feasible, each initial post-injury DETECT assessment was administered on the sidelines during games or practices. In the case of delayed athlete reporting, DETECT was administered in team locker rooms or training rooms.

In addition to testing at baseline and after a suspected concussion, a subset of non-injured, asymptomatic football athletes completed DETECT testing on field sidelines during full contact practice play throughout the season to serve as a non-clinical concussion control group. Athletes were assumed to be asymptomatic if they did not report concussion symptoms to the AT or physician.

### Outcome assessment (diagnosis of concussion)

Definitive diagnosis of a concussion injury was based on the AT's or team physician's final assessment, which was frequently based on an aggregate assessment of symptoms, clinical evaluation, NP evaluation (excluding DETECT), and balance testing, over repeated assessment intervals. A concussion diagnosis by the AT or physician was accepted as a true diagnosis regardless of the concussion assessment protocol. Players who were removed from play under suspicion of concussion injury, but ultimately were determined not to have a concussion injury, were recorded as a distinct cohort from those with a final diagnosis of concussion. ATs, coaches, athletes, and other medical staff remained blinded to DETECT outcomes throughout the data collection period.

### Statistical analysis

Data analysis was performed by researchers who were not involved in subject consent or administration of DETECT. Baseline characteristics of the cohort were summarized descriptively, with frequency and percent for categorical factors and median, interquartile range and range for continuous factors. Baseline characteristics considered to be pre-morbid risk factors of concussion were analyzed using univariate logistic regression models and risk ratios for concussion were reported.

To statistically compare the degree of discrimination between concussed players and non-concussed players, the area under the receiver operating characteristic (ROC) curve (the concordance index) was computed and compared across modalities using established methods.^32^ The main outcome for ROC analysis was composite DETECT score, considered both at time of suspected injury and as change from baseline. Primary analysis was performed on the subset of players who had suspected concussion at time of DETECT assessment. Secondary analysis was done for the entire cohort, wherein players who never had suspected concussion were also included. In addition to composite DETECT score, response time and accuracy were assessed for discriminant ability of concussion for each of the DETECT subtests. Both factors were assessed at time of concussion and as a change from baseline. The Bonferroni method was used to correct for 32 multiple comparisons in the analysis of test subsets.

The reliable change index (RCI) was used to assess change between baseline and in-season DETECT score. The estimate of the standard error of measurement of the difference score was calculated as 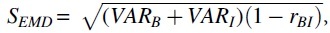 where *VAR_B_* and *VAR_I_* are the variance estimates of the DETECT score for baseline and in-season tests, respectively, and *r_BI_* is the reliability coefficient common to baseline and post-season tests, equal to 0.60. The RCI statistic was calculated as the difference between baseline and in-season DETECT score, divided by the S_EMD_; when the statistic exceeded 1.96 in absolute value, the change was deemed reliable. Reliable change was compared with concussion using a Fisher's exact test to assess association.

Determination of the relationship between linear and rotational acceleration and NP performance was done by calculating the non-parametric Spearman's correlation coefficient and linear mixed model across acceleration range for the mean response time and accuracy for each of the DETECT subtests. Secondary analysis was performed on all hits >50*g*. Sensitivity analysis was performed removing the highest acceleration level for each correlation.

All tests were evaluated for statistical significance at the 0.05 alpha level. Statistical analysis was performed using SAS 9.4 (Cary, NC, USA).

## Results

**FIG. 2.**
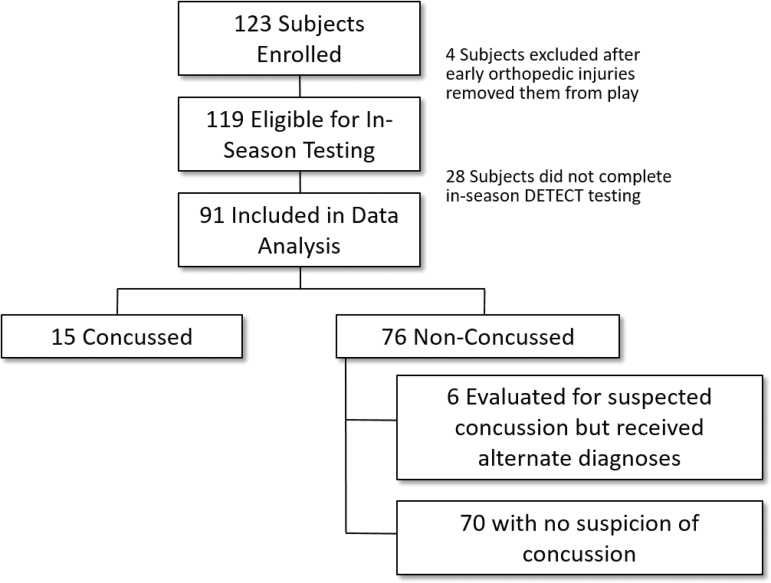
Participant inclusion diagram. A total of 123 athletes were enrolled and completed baseline DETECT testing. Among eligible participants, 91 completed in-season DETECT testing, and were included in concussion analysis. For subconcussive HIT analysis 76 non-concussed players were included. DETECT, Display Enhanced Testing for Cognitive Impairment and mTBI; HIT, Head Impact Telemetry; mTBI, mild traumatic brain injury.

A total of 123 athletes were enrolled and completed baseline DETECT testing. Among eligible participants, 91 completed in-season DETECT testing, and were included in analysis (Fig. 2). Of the 32 who did not complete the study, 5 had season ending injuries, and 27 had either poor effort tests, incomplete tests, or were lost to follow-up. Baseline characteristics for the complete study cohort, as well as for high school and college-level players, are provided in Table 1. Median age was 18 years (range 16–23 years; *n* = 50 < 18 years), and all participants were male. Forty-four percent (*n* = 54) of participants were high school athletes.

**Table 1.** Baseline Characteristics of Cohort, Overall and by Type of Institution

	Overall (*n* = 123)	College players (*n* = 69)	High school players (*n* = 54)
Race^a^ – frequency (%)			
White	85 (69.7%)	37 (54.4%)	48 (88.8%)
Black	29 (23.8%)	25 (36.8%)	4 (7.4%)
Asian	3 (2.4%)	2 (2.9%)	1 (1.9%)
More than one race	5 (4.1%)	4 (5.9%)	1 (1.9%)
Hispanic ethnicity	6 (4.9%)	0 (0%)	6 (11.1%)
History of a previous concussion	54 (43.9%)	30 (43.5%)	24 (44.4%)
Age (years) – median (Q1, Q3) [min-max]	18 (17, 20)	20 (19, 21)	17 (16, 17)
	[16-23]	[18-23]	[16-18]
Height (in)	72 (70, 74)	73 (71, 75)	71 (69, 73)
	[66-80]	[66-80]	[67-78]
Weight (lb)	200 (185, 235)	220 (200, 254)	182.5 (170, 200)
	[122-319]	[165-319]	[122-264]
BMI	27.6 (25.1, 30.8)	29.4 (27.5, 32.9)	25.0 (23.7, 27.3)
	[18.5-40]	[24.4-40]	[18.5-31.6]
Number of years of collision sport play^b^	10 (8, 11)	10 (8, 14)	9.5 (7, 10)
	[1-18]	[1-18]	[4-14]
Baseline DETECT score	2.0 (1.5, 2.7)	2.1 (1.6, 2.8)	1.9 (1.5, 2.4)
	[1.2-10.0]	[1.3-9.4]	[1.2-10.0]

^a^ One college player did not report race.

^b^ Missing for 2 college players.

BMI, body mass index; DETECT, Display Enhanced Testing for Cognitive Impairment and mTBI; mTBI, mild traumatic brain injury.

Twenty-one athletes sustained impacts concerning for concussion over the course of a single season. Median time from suspected injury to DETECT testing was 15 min (range 1–282 min) among athletes available for sidelines testing (*n* = 14). Of the remaining 7 athletes completing DETECT testing for suspected concussion, 1 player did not have a time of injury recorded, and 6 were tested within 1 to 5 days of suspected injury due to delayed onset or delayed reporting of symptoms. Fifteen players were ultimately diagnosed with a concussion based on the institutions' standard concussion assessment protocols. Of these, 9 were from the “immediately removed” group and 6 were from the “delayed onset” group.

### DETECT outcomes

Among the 21 players tested for suspected concussion, a composite DETECT score of 1.59 (range of 0–10, with higher values indicating greater cognitive impairment) measured at the time of suspected concussion (rather than change from baseline, which is reported below) was 86.7% sensitive (13 of 15 concussed players correctly identified; 95% confidence interval [CI]: 59.5%, 98.3) and 66.7% specific (4 of 6 non-concussed players correctly identified; 95% CI: 22.3%, 95.7%) for identifying post-impact cognitive impairment (Table 2). Within this high-risk group, ROC analysis demonstrated DETECT significantly discriminates between concussed and non-concussed players (area under the curve [AUC] 0.778, 95% CI: 0.54, 1.0, *p* = 0.02).

**Table 2.** DETECT Outcomes

Cohort	AUC (95% CI)	*P*-value	Sensitivity (95%CI)	Specificity (95% CI)
Composite DETECT score				
Suspected concussion only (*n* = 21)	0.778 (0.544, 1.0)	0.02	86.7% (59.5, 98.3)	66.7% (22.3, 95.7)
All players (*n* = 91)	0.727 (0.586, 0.86)	0.002	86.7% (59.5, 98.3)	43.4% (32.1, 55.3)
Change from baseline				
Suspected concussion only (*n* = 21)	0.778 (0.575, 0.980)	0.007	66.7% (38.4, 88.2)	66.7 % (22.3, 95.7)
All players (*n* = 91)	0.716 (0.549, 0.882)	0.01	66.7% (38.4, 88.2)	59.2% (47.3, 70.4)

AUC, area under the curve; CI, confidence interval; DETECT, Display Enhanced Testing for Cognitive Impairment and mTBI; mTBI, mild traumatic brain injury.

When players with and without diagnosed concussion were considered (*n* = 85), the ability of DETECT to distinguish between concussed and non-concussed players remained significant (AUC 0.73, *p* = 0.002) with maintained sensitivity 86.7% (13 of 15 concussed players correctly identified; 95% CI: 59.5%, 98.3%), although specificity was reduced (33 of 70 players not suspected of concussion correctly identified; 43.4%; 95% CI: 32.1%, 55.3%) due to the relatively small number of concussions in this cohort (Table 2).

### Change in DETECT from baseline testing

Change from baseline was not associated with concussion when using an RCI (Fisher's exact *p* = 0.19). Among 21 players evaluated for suspicion of concussion, a change in the DETECT score from baseline demonstrated a statistically significant yet clinically limited ability to discriminate between concussed and non-concussed players (10 of 15 concussed players correctly identified; sensitivity 66.7%, 95% CI: 38.4%, 88.2%; 4 of 6 non-concussed players correctly identified; specificity 66.7, 95% CI: 22.3%, 95.7%; AUC 0.778, 95% CI: 0.575 – 0.980, *p* = 0.007; Table 2).

When players with and without diagnosed concussion were considered (*n* = 85), the change in DETECT score from baseline distinguished between concussed and non-concussed players (AUC 0.719, *p* = 0.01, 95% CI: 55.4%, 88.4%) with maintained sensitivity 66.7% (10 of 15 concussed players correctly identified; 95% CI: 38.4%, 88.2%), although specificity was reduced (42 of 70 players not suspected of concussion correctly identified; 95% CI: 47.6%, 71.5%) due to the relatively small number of concussions in this cohort (Table 2).

### Performance on DETECT subtests among concussed players

Accuracy and/or response time during conditional choice, complex choice, and delayed word recall subtests demonstrated significant discriminant ability between concussed and non-injured athletes. Longer reaction time in the conditional choice subtest, as change from baseline, showed significant discriminant ability to identify concussed players (AUC 0.811, 95% CI: 0.619, 1; *p* = 0.001). Moreover, lower accuracy in this task at the time of suspected injury showed significant discriminant ability as well (Table 3). Similarly, complex choice reaction time showed significant discriminant ability to identify concussions (AUC 0.956, 95% CI: 0.86, 1; *p* < 0.001). Accuracy on this task also had discriminate ability, both at the time of suspected injury (AUC 0.822, 95%CI: 0.666, 0.978; *p* < 0.001) and as change from baseline performance (AUC 0.928, 95% CI: 0.818, 1; *p* < 0.001). Performance on delayed word recall was also a significant discriminate factor, both at time of sidelines testing (AUC 0.878, 95% CI: 0.726, 1; *p* < 0.001) and when change from baseline was considered (AUC 0.919, 95% CI: 0.79, 1; *p* < 0.001). Immediate word recall and N-back were not discriminatory between groups. 

**Table 3.** Mean Response Time and Accuracy of DETECT Subtests, at Time of Concussion and Change from Baseline (for Diagnosis of Concussion in Players with Suspected Concussion, *n* = 21)

	At time of suspected concussion	Change from baseline
Mean response time	AUC (95% CI), P	
Conditional choice	62.2% (36.3%, 88.1%); 0.36	**81.1% (61.9%, 100%); 0.001**
1-Back	60.0% (31.7%, 88.3%); 0.49	56.7% (30.1%, 83.2%); 0.62
2-Back	56.7% (28.1%, 85.2%); 0.65	44.4% (18.9%, 70.0%); 0.67
Complex choice	53.3% (28.3%, 78.3%); 0.79	**95.6% (86.0%, 100%); <0.001**
Immediate word recall	53.3% (25.4%, 81.2%); 0.82	55.6% (24.8%, 86.3%); 0.72
Selective reminding	55.6% (28.5%, 82.7%); 0.69	53.3% (25.5%, 81.1%); 0.81
Delayed word recall	62.2% (32.6%, 91.8%); 0.42	75.6% (45.8%, 100%); 0.09

Accuracy	AUC (95% CI), P	
Conditional choice	**85.6% (71.0%, 100%); <0.001**	50.0% (26.0%, 74.0%); 0.99
1-Back	65.6% (40.7%, 90.4%); 0.22	61.7% (38.7%, 84.7%); 0.32
2-Back	75.0% (48.4%, 100%); 0.07	65.0% (40.3%, 89.7%); 0.23
Complex choice	**82.2% (66.6%, 97.8%); <0.001**	**92.8% (81.8%, 100%); <0.001**
Immediate word recall	59.4% (28.3%, 90.6%); 0.55	49.4% (20.6%, 78.3%); 0.97
Selective reminding	56.7% (32.2%, 81.1%); 0.59	63.3% (35.9%, 90.7%); 0.34
Delayed word recall	**87.8% (72.6%, 100%); <0.001**	**91.1% (79.0%, 100%); <0.001**

Bolded text indicates significant discriminatory ability of the subtest to identify concussed players.

AUC, area under the curve; CI, confidence interval; DETECT, Display Enhanced Testing for Cognitive Impairment and mTBI; mTBI, mild traumatic brain injury.

### Reliable change index (RCI)

In RCI analysis, both players with significant increases and decreases in DETECT score from baseline to in-season were identified as having reliable change. Five players were determined to have reliable change (2 with worse in-season scores compared with baseline and 3 with an improved score). Reliable change was not significantly associated with concussion (Fisher's exact *p* = 0.19). A small subset of players with abnormally high baseline scores (*n* = 2, scores >9) drove inflation of variance estimates and as a result, very few players were identified as having a reliable change. In a sensitivity analysis with these 2 players removed, 15 players were determined to have reliable change (5 with a lower in-season score compared with baseline and 10 with an improved score) and reliable change was also not significantly associated with concussion (Fisher's exact *p* = 0.24).

### Head impact descriptive outcomes

Over the season, 34,679 total impacts (12,091 in high school players and 22,589 in collegiate players) were recorded, with 35% of impacts occurring during competition events and 65% occurring during practices. The impact locations on the helmet were as follows: 38% front, 27% top, 22% back, and 13% to the side, indicating that the majority (60%) of impacts likely resulted in sagittal plane motion. Linear acceleration ranged from 30*g* (set threshold) to 192*g* and rotational acceleration ranged from 602 rad/sec^2^ to 12,115 rad/sec^2^. There was no difference in average linear acceleration or average rotational acceleration between competition (39.1 ± 22.6*g* and 2367 ± 1633 rad/sec^2^, respectively) and practice (38.9 ± 21.3*g* and 2154 ± 1425 rad/sec^2^, respectively). Video analysis of four games found that 11% of hits were false-positive, consistent with other reports,^33^ and with the error range found when comparing HIT with Hybrid III instrumented headforms during laboratory tests.^34,35^

### Neuropsychological performance and head impact relationship

**FIG. 3.**
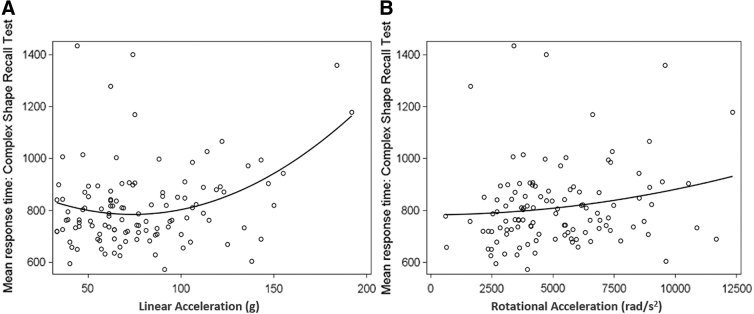
Correlation of helmet impact acceleration to neuropsychological performance in players without concussion. Complex choice reaction time significantly correlated with helmet acceleration. **(A)** Mean reaction time significantly increases with increase in linear acceleration (Spearman's coefficient 0.262; *p* = 0.02), and **(B)** rotational acceleration (Spearman's coefficient 0.254 , *p* = 0.03); *n* = 113 tests on 76 players.

A total of 113 DETECT tests were done on 76 players, none of whom had concussive signs or symptoms at the time of testing. Increased response time on the complex choice reaction time test positively correlated with both linear (Spearman's rank correlation coefficient 0.231, *p* = 0.04) and rotational acceleration (Spearman's coefficient 0.224, *p* = 0.05). Sensitivity analysis with removal of potential outlier acceleration maintained significance for linear (Spearman's coefficient 0.262, *p* = 0.02) and rotational (Spearman's coefficient 0.254, *p* = 0.03) acceleration (Fig. 3). Although complex choice reaction time consistently showed a correlation, the other subtests were not significantly correlated with helmet acceleration.

**FIG. 4.**
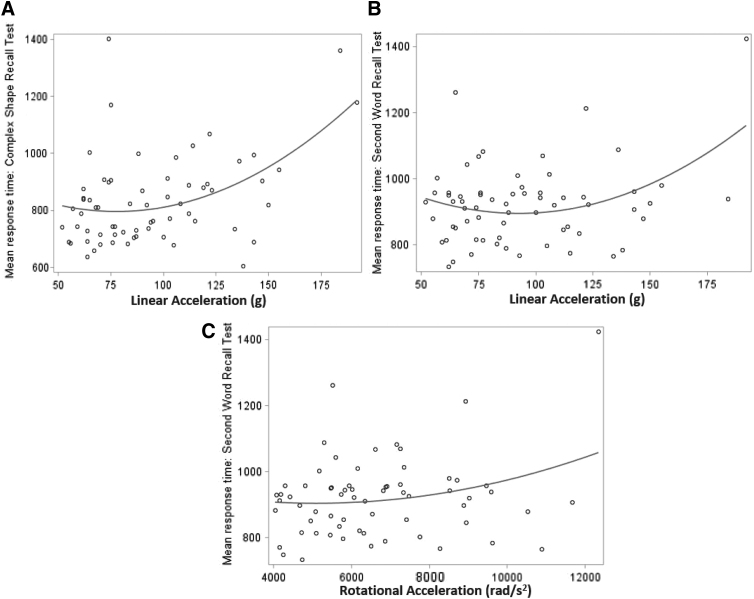
Helmet impact acceleration correlation to neuropsychological performance in players without concussion with linear acceleration >50*g* and 4000 rad/sec^2^. **(A)** Complex choice mean reaction time significantly correlated with linear acceleration Spearman's coefficient 0.267, *p* = 0.05; univariate mixed model, *p* = 0.03. **(B)** Selective reminding visual word recall mean reaction time significantly correlated with increases in linear acceleration (univariate mixed model, *p* = 0.05). **(C)** Rotational acceleration (univariate mixed model, *p* = 0.05); *n* = 56 players.

Secondary analysis of linear accelerations greater than 50*g* and rotational accelerations greater than 4000 rad/sec^2^ (66 tests on 56 players) showed a significant correlation between complex choice reaction time and linear acceleration (Spearman's coefficient 0.267, *p* = 0.05; univariate mixed model, *p* = 0.03). Using a univariate linear mixed model revealed significant relationships between complex choice reaction time and linear acceleration (*p* = 0.03), as well as between selective reminding response time and linear (*p* = 0.05) and rotational (*p* = 0.05) acceleration (Fig. 4). The other DETECT tests did not show correlation with linear or rotational acceleration for response time or accuracy in this cohort.

## Discussion

In the present study, we found DETECT confers moderate to high sensitivity in identifying acute cognitive impairment associated with concussion when compared with a medical professional's final summative clinical diagnosis of concussion. Of note, DETECT results were collected at the time of suspected injury, and independent of clinical assessment, to avoid the inclusion bias that is present from other test modalities. In this respect, NP assessment with DETECT alone was 87% sensitive in identifying clinically concussed individuals at the time of injury, although specificity was lower at 66.7%. A direct head-to-head comparison of DETECT with other concussion assessments is of future interest, but was not the goal of the current study.

In the analysis of the individual DETECT subtests, we report significant differences between concussed and control subjects in conditional choice reaction time, complex choice reaction time, and delayed word recall. Moreover, these differences were evident both at the time of injury and when compared with pre-season baseline scores. Choice reaction time tests measure recognition memory, requiring a decision on more than one feature to be made prior to responding and have been shown to be sensitive for concussion in previous studies.^36,37^ Conditional choice further increases cognitive load over complex choice, because there is a condition on the correct response (i.e., indicate the direction in which the arrow is pointing if it is a particular color, otherwise select the opposite direction). In addition, delayed recall taxes both working memory and attention and has been found to be sensitive in detecting concussion.^38,39^ Overall, as cognitive load increased (i.e., complex choice < conditional choice < delayed recall) accuracy showed more discriminating ability than response time, which is consistent with the long-standing speed-accuracy trade-off phenomenon.^40^ By covering multiple neurocognitive domains and difficulties, DETECT may be able to identify a range of concussion phenotypes.

All study participants, irrespective of concussion status, completed baseline testing prior to the beginning of the football season. We did not find a change from baseline to be clinically useful in discriminating between concussed and non-concussed individuals. Using a change of −0.011, comparison of DETECT performance from baseline was only 66.7% sensitive and 60.0% specific for concussion. Although these data were statistically discriminatory (AUC 0.719, *p* = 0.01), we did not find incremental change from baseline to be sufficiently sensitive to merit its use as an initial screening metric for acute neurological injury. Moreover, we did not find an RCI to be associated with clinical concussion.

Other authors have reported similar results with respect to baseline testing.^41–43^ When comparing post-concussion performance on ImPACT assessment with either change from baseline or deviation from population norms, the proportion of college athletes demonstrating a decline from baseline was no greater than what would be expected to occur from chance alone.^42^ In a more comprehensive evaluation of baseline testing no difference was found between a baseline change approach and a normative comparison approach in identifying post-concussive deficits using a symptoms checklist or postural stability assessments; whereas the data provided conflicting results for computerized neurocognitive testing.^43^

Although an extensive review of empirical data on baseline testing has been reported elsewhere^41,44^ and is beyond the scope of discussion, it is important to note that the Concussion in Sport Group no longer recommends routine use of baseline NP testing.^15^ The NCAA however continues to endorse a “one-time, pre-participation baseline concussion assessment for all varsity student athletes” including NP testing. Thus, although consensus guidelines remain somewhat in conflict, emerging data suggest point-of-care testing at the time of suspected injury may be a reasonable and more clinically appropriate approach to identifying concussion.

Interestingly, when we tested all football players after a known impact level and with no concussion diagnosis, two DETECT subtests were able to identify cognitive deficits as a function of linear and rotational impact acceleration, albeit with relatively weak correlation. These results suggest that football impacts that do not result in concussion may negatively affect cognitive performance immediately follow an impact. Specifically, deficits in acute processing speed and divided attention (complex choice reaction time) correlate with impact acceleration across a wide range of accelerations.

For a subset limited to higher level impacts (>50*g*, >4000 rad/sec^2^), processing speed and divided attention (complex choice reaction time) deficits correlate with linear acceleration, whereas working memory deficits (selective reminding word recall) correlate with both linear and rotational acceleration. There have been other similar observations of subclinical or subconcussive impairments. Repeat head impact variables in collegiate athletes measured with HIT correlated with deficits in visual memory in women soccer players and with King-Devick in football.^45^ Similarly, visual working memory deficits as measured with ImPACT were observed in high school football players without clinically diagnosed concussion.^46^ In a virtual reality platform, collegiate football athletes had significant deficits in spatial navigation but not balance or reaction time as a function of the number and level of impacts over a season.^47^ In addition, functional magnetic resonance imaging (fMRI) signal changes across nearly one-third of 116 regions of interest were shown to correlate with the number of impacts in both concussed and non-concussed football players,^48^ suggesting that cumulative impacts may affect network function.^49^ Similarly, white matter changes, as detected with diffusion tensor imaging (DTI) correlate with impact measures.^50,51^ Certainly, recent attention highlights the need to further investigate the neurological and biomechanical features associated with subconcussive, repetitive impacts in athletic activity.^52–54^

In terms of feasibility, we found that high school teams had more flexibility then collegiate teams. Removing players from practice, for example, was easier to do with high school teams versus college, with clear communication between the coaching staff and researchers a key to success (e.g., removal for testing occurs at next break or at 15 min post impact-of-interest, whichever is sooner). Athletes were willing to be tested on the sidelines and were able to pay attention to the tests, based on very few tests lost to poor effort and incompletion (∼6% total tests). This is consistent with a pilot study we conducted, in which there were no differences in DETECT scores with administration in a quiet room versus a room with simulated stadium noises (data not published). Not all teams we initially approached were willing to participate in the study and this certainly points to the need for more education about research within athletic programs as well as for investigators navigating research studies with athletes and athletic staff.

We acknowledge several limitations to this study. Primarily, the limited number of concussions in our study cohort increases the risk of a sampling error during analysis of DETECT sensitivity and specificity for identifying post-traumatic cognitive impairment. We attempted to mitigate this risk *a priori* by increasing the sample size and combining injury data from four separate football teams. Although the frequency of concussion observed in our study was quite high (16.4%), nonetheless we recognize further research is warranted to assess and improve the discriminatory power of the DETECT outcome score.

A second limitation to our study includes the use of football athletes participating in full-contact play to serve as controls. The decision to include active football players as subject controls was made to limit environmental (e.g., temperature), clinical (e.g., timing of assessment, level of dehydration), and methodological (e.g., access to athletes, level of distracting factors during testing) variables that could influence cognitive outcomes. Although none of the control athletes displayed signs or symptoms consistent with concussion, they were certainly exposed to repetitive head impacts prior to and throughout the study period, which may have reduced or otherwise influenced their DETECT performance. Indeed, we show that subconcussive impacts may affect cognitive performance immediately following impact and recognize the need for expanded studies with a true non-impact athletic control group.

Despite these limitations, DETECT provides a clinically relevant, and statistically significant solution for point-of-care NP testing in athletic environments. Further, the ability to complete the DETECT assessment battery in 10 min may be an important advantage for acute triage and return-to-play decisions. Further investigation is warranted to determine the role of DETECT NP testing in combination with other clinical assessments of concussion injury, such as balance and oculomotor function as recommended by the Consensus Statement of the International Conference on Concussion in Sport.^15^ Future directions will include expansion of DETECT to include additional modalities, while optimizing subtest inclusion based on test duration, as well as sensitivity and specificity.

## Conclusion

DETECT is a unique neurocognitive assessment tool designed to address some of the limitations of other clinical evaluation tools to improve the assessment and care of potentially injured athletes acutely following suspected concussion. The immersive environment is novel and expands the ability to assess athletes in distracting situations such as sidelines, as well as other situations where prompt objective cognitive triage is needed. In this modest cohort of competitive high school and collegiate football athletes, DETECT provided moderate to high sensitivity and discriminatory value in identifying cognitive impairment acutely following a concussion. Several NP subtests within DETECT show promise for identifying concussion in the absence of baseline testing. Expansion to other neurological domains is expected to improve both sensitivity and specificity of the test and future studies will include comparison with other concussion assessment tools using a larger subject size. Used in combination with impact sensors, multiple neurocognitive domains can be assessed as a function of impact measures, providing a sensitive tool to examine the effect of repetitive impact in the absence of clinical concussion.

## Abbreviations Used

AT: athletic trainer
AUC: area under the curve
CI: confidence interval
DETECT: Display Enhanced Testing for Cognitive Impairment and mTBI
DTI: diffusion tensor imaging
ED: emergency department
fMRI: functional magnetic resonance imaging
HIT: Head Impact Telemetry
ImPACT: Immediate Post-Concussion Assessment and Cognitive Testing
IRB: Institutional Review Board
MCI: mild cognitive impairment
MMSE: Mini Mental State Examination
mTBI: mild traumatic brain injury
NCAA: National Collegiate Athletics Association
NP: neuropsychological
RCI: reliable change index
ROC: receiver operating characteristic
SCAT: Sport Concussion Assessment Tool
SRC: sports related concussion
